# Physicochemical and biological characterization of a bispecific antibody in a CrossMab/KIH format that targets EGFR and VEGF-A

**DOI:** 10.3389/fimmu.2025.1659966

**Published:** 2025-09-03

**Authors:** Safiat Ayinde, Shraboni Dutta, Nishant Mohan, Wen Jin Wu

**Affiliations:** Division of Pharmaceutical Quality Research III, Office of Pharmaceutical Quality Research, Center for Drug Evaluation and Research, U.S. Food and Drug Administration, Silver Spring, MD, United States

**Keywords:** bispecific antibody (BsAb), CrossMab, knobs-into-holes (KiH), epidermal growth factor receptor (EGFR), vascular endothelial growth factor A (VEGF-A), ovarian cancer (OC)

## Abstract

**Introduction:**

Bispecific antibodies (BsAbs) are a class of antibody therapeutics engineered in various molecular formats to bind two distinct antigens and potentially mediate multiple biological effects. These molecular formats are tailored to mediate specific mechanisms of action and possess unique physicochemical and biological properties that are necessary to assure product quality. In ovarian cancer (OC), both EGFR- and VEGF-A-mediated signaling pathways are often upregulated and cooperate to promote tumor growth and angiogenesis. Thus, inhibiting of EGFR- and VEGF-A pathways with a BsAb may provide synergistic anti-tumor activity.

**Methods:**

Using publicly available sequences and applying immunoglobulin domain crossover (CrossMab) and knobs-into-holes (KIH) technologies, we generated a BsAb to simultaneously bind EGFR and VEGF-A (designated as anti-EGFR/VEGF-A BsAb). This BsAb served as a model for physiochemical and biological characterization of quality attributes that would be critical for the BsAb’s mechanisms of action. Our goal was to gain fundamental insights into BsAbs designed to target a receptor with one arm and a soluble ligand with the other, to support bioassay development and inform quality control strategies.

**Results:**

Our data demonstrated that the CrossMab/KIH platform successfully produced a correctly assembled BsAb during cell culture. Characterization confirmed that the anti-EGFR/VEGF-A BsAb bound both EGFR and VEGF-A with comparable activity and affinity to the respective parental monoclonal antibodies. Functionally, the BsAb disrupted both EGF/EGFR and VEGF-A/VEGFR2 signaling pathways in OC and human umbilical vein endothelial cell (HUVEC) models. Furthermore, the BsAb effectively blocked angiogenic signaling driven by VEGF-A secreted from OC cells in a paracrine manner.

**Discussion:**

Based on the combinatorial mechanism of action and our characterization findings, we concluded that two or more bioassays may be needed to accurately assess the activity of both arms of this type of BsAb.

## 1 Introduction

Bispecific antibodies (BsAbs) possess two binding sites that bind to two specific antigens or two different epitopes in the same antigens (1, 2). In the past two decades, genetic engineering to design different molecular formats of BsAbs has overcome many technical hurdles to ensure correct pairing of heavy and light chains of BsAb (3). These formats of BsAbs are designed to allow binding to two proteins expressed on either the same or the different cells, two different ligands/cytokines, or one ligand/cytokine and one receptor, and have the unique potential to mediate the proposed mechanism(s) of action for the intended clinical indications and may have advantages as compared to monoclonal antibodies (mAbs) that bind a single antigen (1–5). Thirteen BsAbs have been approved for use in cancer, hematological, and ocular disease treatments, and over one hundred bispecific antibodies are currently being tested in clinical trials (5–10). The number of approaches to generate genetically engineered BsAbs have greatly expanded over time. CrossMab technology has emerged as a versatile, reliable approach to engineering recombinant bi- and multi-specific antibodies and fusion proteins (3, 11–15). As of mid-2021, at least 19 BsAbs and fusion proteins based on CrossMab technology have entered clinical trials (16). Faricimab (FAR) is an example of a successful CrossMab BsAb approved for the treatment of diabetic macular edema, neovascular wet-aged macular degeneration, and macular edema following retinal vein occlusion (17). FAR treatment has been shown to have a longer-lasting effect than single anti-VEGF agents through the simultaneous inhibition of VEGF-A and Ang-2 (17). Overall, the utilization of a BsAb with a CrossMab format has the potential to facilitate development of therapeutic agents for more complex diseases that develop resistance or have high recurrence rates, such as ovarian cancer.

Ovarian cancer (OC) is the deadliest among gynecologic cancers with a 50.8% 5-year survival rate (18). The current standard of care consists of surgery followed by adjuvant chemotherapy, radiation therapy, immunotherapy, or targeted therapy (19). However, approximately 50-70% of the patients will experience recurrence (20). Despite recent progress, advanced OC remains a disease of high unmet need. Epidermal growth factor receptor (EGFR) and vascular endothelial growth factor A (VEGF-A) have been shown to contribute to cancer cell survival and have been identified as potential therapeutic targets for advanced OC (21, 22). EGFR promotes cell growth, survival and chemoresistance, and up to ~70% of OC patients are EGFR positive (21). Unfortunately, targeting EGFR alone has shown disappointing clinical results (21). VEGF-A promotes angiogenesis to improve cell invasion and metastasis (22). OC secretes large amount of VEGF-A *in vitro* and *in vivo* (23, 24). Targeting VEGF-A with bevacizumab has shown some promise clinically when combined with chemotherapy, but most patients relapse during or after bevacizumab treatment developing drug resistance (25). Co-targeting EGFR and VEGF-A/VEGFR2 could synergistically amplify anticancer activity of targeted therapy (26). This co-targeting approach has shown promise in *in vitro* cancer models, including OC and triple negative breast cancer (TNBC) with an anti-EGFR/VEGFR2 BsAb (27, 28).

This study aimed to generate a BsAb targeting EGFR and VEGF-A using CrossMab/knobs-into-holes (KIH) technologies. We used this BsAb as a model to characterize its critical quality attributes (CQAs) and develop bioassay(s) to assess its anti-tumor activity/potency that reflects mechanisms of action of the product. Additionally, data from this study provided insights into the roles of EGFR and VEGF-A/VEGFR2 signaling pathways in OC, aiding in the development of novel therapeutic drugs to treat OC.

## 2 Materials and methods

### 2.1 Cell culture and reagents

The OC cell lines, CaOV3, SKOV3, OVCAR3, PA-1, were purchased from American Type Culture Collection (ATCC Manassas, VA, USA) and maintained in cell culture media, fetal bovine serum, and supplements recommended by ATCC. The adherent human umbilical vein endothelial cells (HUVEC) were purchased from ScienCell (Carlsbad, CA, USA) and ATCC (Manassas, VA, USA). Both HUVEC cell lines were propagated in endothelial culture media supplemented with growth factors and bovine plasma fibronectin as instructed by each vendor. Therapeutic monoclonal and bispecific antibodies cetuximab, ramucirumab, bevacizumab, and faricimab were purchased from WEP clinical (Morrisville, NC). The EGF, EGFR, and VEGF-A proteins were obtained from Raybiotech (Peachtree Corners, GA, USA). Biotinylated human EGFR protein, His,Avitag™ was obtained from Acro Biosystems (Newark, DE, USA). Horseradish peroxidase (HRP)-conjugated streptavidin was obtained from Thermo Fisher Scientific (Waltham, MA, USA).

### 2.2 Construction, expression, and purification of the anti-EGFR/VEGF-A CrossMab/KIH BsAb

The faricimab heavy chain knob, faricimab light chain, cetuximab heavy chain hole light chain CL crossed, and cetuximab light chain CH1 crossed sequences were derived from a publicly available database, International Immunogenetics Information System (http://www.imgt.org). The cetuximab light chain was designed as a CrossMab CH1–CL orientation, and the cetuximab and faricimab heavy chains were designed as a knobs-into-holes (KIH) orientation. The genes were chemically synthesized and then cloned into pCDNA3.4 (+) between the EcoRI and HindIII sites and confirmed by sequencing (27). Four plasmids encoding heavy and light chains of anti-EGFR/VEGF-A BsAb were transiently transfected into mammalian HEK 293 cells. The methods for the generation of plasmid constructs were similar to that as previously described (27). The anti-EGFR/VEGF-A BsAb was assembled during cell culture. The purification and elution of anti-EGFR/VEGF-A BsAb was performed by affinity chromatography using MabSelect™ PrismA Resin+ Prism G Resin FF.

### 2.3 SDS-PAGE analysis

Two µg of cetuximab, faricimab, or anti-EGFR/VEGF-A BsAb samples were prepared in non-reducing and reducing conditions. All samples were subjected to 4–15% gradient SDS-PAGE. After separation, gels were incubated in Simply Blue Coomassie stain at room temperature until saturated. Then, gels were washed in water to remove excess stain. The antibody bands were visualized by ChemiDoc MP gel imaging system (BioRad, Hercules, CA, USA).

### 2.4 CE-SDS analysis

The anti-EGFR/VEGF-A BsAb and faricimab samples along with an IgG standard were studied in reducing conditions using the Maurice CE-SDS PLUS method (ProteinSimple, San Jose, CA) as per the manufacture’s recommendations. The CE-SDS experiments in non-reducing conditions were performed as previously reported (34). For reduced conditions, 2.5 μL of 14.2M β-mercaptoethanol was added to the samples and IgG standard. The samples and IgG standards were transferred to a 96-well plate that was placed inside the Maurice system for analysis. The samples and IgG standards were injected onto a CE-SDS PLUS Cartridge at 4.6 kV for 20 s. The reduced samples were separated at 5.75 kV for 45 min, and the non-reduced IgG standard was separated at 5.75 kV for 35 min. Data were collected using Compass for iCE Version 4.0.0 software (ProteinSimple, San Jose, CA).

### 2.5 SEC-HPLC

HPLC experiments were performed as previously reported (34).

### 2.6 ELISA assay

A standard ELISA assay was performed to detect the binding of anti-EGFR/VEGF-A BsAb and cetuximab to EGFR and the binding of anti-EGFR/VEGF-A BsAb, faricimab, and bevacizumab to VEGF-A as described previously (27). In addition, an ELISA assay was performed to detect the simultaneous binding of anti-EGFR/VEGF-A to VEGF-A and biotinylated human EGFR. The protocol was similar to the standard ELISA assay as described previously (27). A 96-well plate was coated with 1 µg/mL of VEGF-A overnight at 4°C and then were washed and blocked with 5% BSA. Following an additional wash, anti-EGFR/VEGF-A BsAb was added. The wells were then washed again followed by addition of 2 µg/mL of biotinylated human EGFR to the 96-well plate. After incubating overnight at 4°C, the wells were washed with 1X PBST, and HRP-conjugated streptavidin was added to the 96-well plate. The remaining steps were performed as previously described (27).

### 2.7 Biacore binding kinetics assay

Surface plasmon resonance (SPR)measurements were performed using a Biacore T200 optical biosensor instrument (GE Healthcare, Piscataway, NJ, USA) to detect the binding kinetics of anti-EGFR/VEGF-A BsAb to EGFR and VEGF-A as described previously (27). The data were processed and fitted to a 1:1 Langmuir binding model using the Biacore 8K Evaluation Software 3.0.

### 2.8 Western blot analysis

Western blot analysis was performed as previously reported (27, 34). Briefly, cells were subjected to serum starvation overnight and then pre-treated with indicated monoclonal and bispecific antibodies (10 μg/mL). After antibody treatment, cells were exposed to EGF, VEGF-A or EGF +VEGF-A for 15 min at a concentration of 100 ng/mL. After treatment, cells were lysed in buffer containing NP-40 to prepare WCL, and the samples were subjected to western blot analysis. The primary antibodies directed against EGFR, phospho-EGFR, VEGFR2, phospho-VEGFR2, FAK, phospho-FAK, Akt and phospho-Akt were purchased from Cell Signaling Technology (Danvers, MA, USA). The GAPDH antibody was purchased from Thermo Fisher Scientific (Waltham, MA, USA). The actin antibody and HRP-conjugated secondary antibodies were purchased from Sigma-Aldrich (St. Louis, MO, USA).

### 2.9 VEGF activity bioassay

The VEGF Activity Bioassay was purchased from Promega (Madison, MI, USA) and was performed as per the manufacturer’s recommendations. Briefly, KDR/NFAT-RE HEK293 cells, from a genetically engineered cell line, were incubated with serial dilutions of VEGF-A for 6-h followed by addition of Bio-Glo Reagent to the cell culture. Luminescence was then quantified using a Promega GloMax Discover plate reader (Madison, WI, USA). For the antibody blockade assay, KDR/NFAT-RE HEK293 cells were incubated with serial dilutions of ramucirumab, bevacizumab, faricimab, or the anti-EGFR/VEFG-A BsAb in the presence of VEGF-A (375 ng/mL) for 6 h. Bio-Glo™ Reagent was then added, and luminescence was quantified using a Promega GloMax Discover plate reader. Data were analyzed using Microsoft Excel and GraphPad Prism.

### 2.10 VEGF secretion bioassay

Human VEGF ELISA Kit Picokine^®^ (Cat# 0593) was purchased from Boster Bio (Pleasanton, CA, USA) and was used to quantitate the VEGF secreted from human OC cells in cell culture media. The assay was performed as per the manufacturer’s instruction. SKOV3, PA-1, OVCAR3, or CaOV3 cells were seeded. After reaching approximately 80-90% confluency per well, the cells were serum-starved for 24 h, and the cell culture media then was collected. 100 µL of collected serum-free media were subjected to VEGF ELISA assay/Picokine^®^ to determine the levels of VEGF secreted in the cell culture media.

### 2.11 CellTiter-Glo luminescent cell viability assay

The assay was performed according to manufacturer’s instructions (Promega cat# 7570). Briefly, 10,000 cells were seeded in white bottom 96-well plates and allowed to adhere overnight in media with 1% FBS. After treatment with 10 μg/mL cetuximab, bevacizumab, or anti-EGFR/VEGF-A BsAb, CellTiter-Glo reagent was added to the plates, and luminescence was measured using the Promega Glomax Discover plate reader.

### 2.12 ADCC assay

The ADCC Reporter Bioassay was performed as previously reported (27). Briefly, target cells (10,000 cells/well) were seeded in a white-bottom 96-well plate in 100 μL/well of ADCC Bioassay buffer provided in the manufacturer’s kit. The next day, serially diluted antibodies (ranging from 0.02 μg/mL to 10 μg/mL) were added into wells. ADCC effector cells provided in the kit were prepared per the manufacturer’s instructions and then added to the tumor cells at an effector to target ratio of 6:1. Bio-Glo™ Luciferase Assay Reagent was added to the plates after 5–6 h of incubation in the cell culture incubator. Luminescence was then measured using the GloMax Discover plate reader.

## 3 Results

### 3.1 Generation of an anti-EGFR/VEGF-A BsAb in CrossMab KIH format

**Figure 1:**
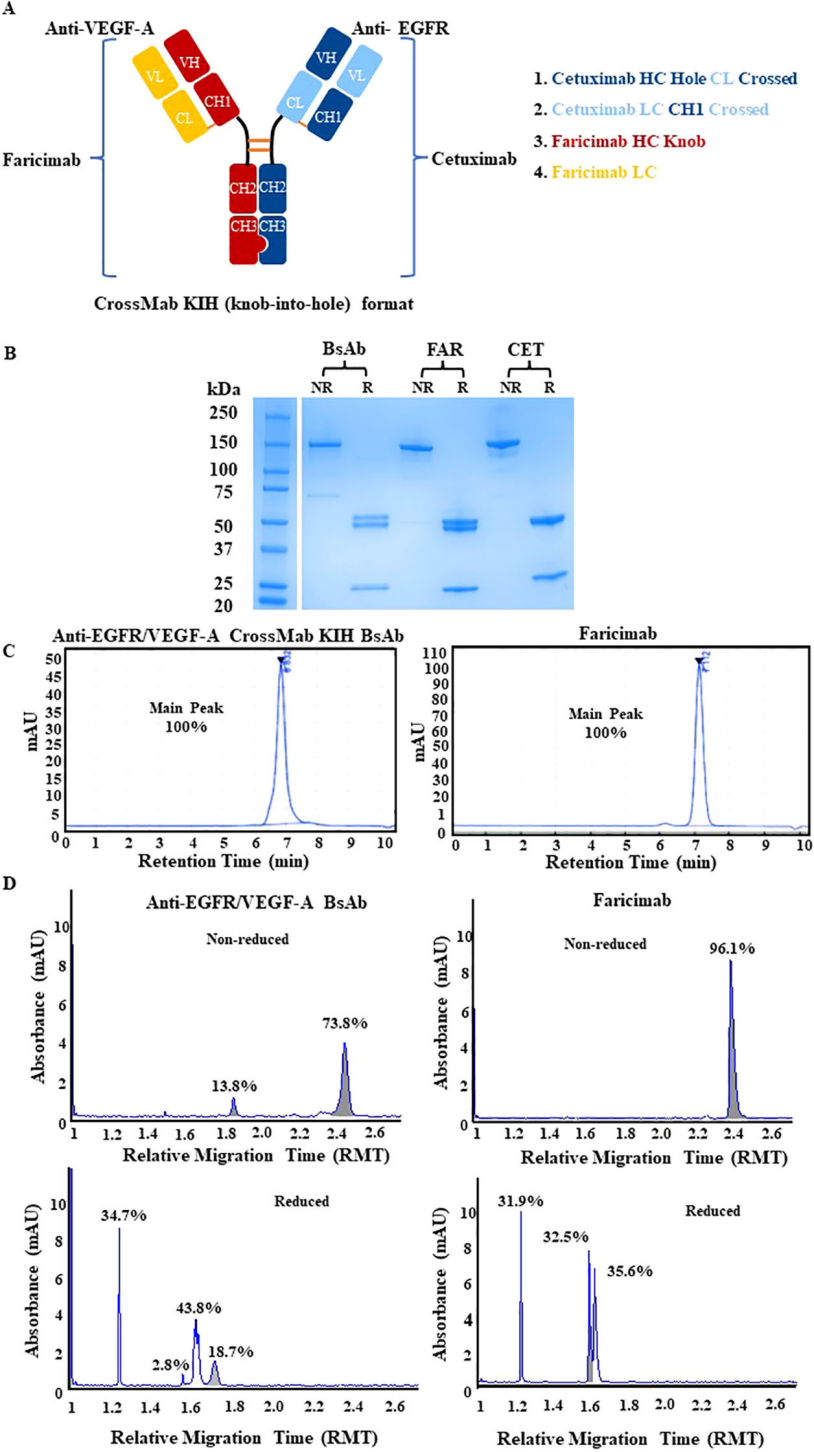
Physiochemical characterization of anti-EGFR/VEGF-A CrossMab/KIH BsAb. **(A)** The schematic representation of anti-EGFR/VEGF-A BsAb with CrossMab/KIH format illustrates how the BsAb was constructed from four antibody fragments derived from faricimab and cetuximab and was designed to target EGFR and VEGF-A. **(B)** Structural integrity was evaluated via SDS-PAGE analysis of 2 µg anti-EGFR/VEGF-A BsAb, 2 µg faricimab (FAR), and 2 µg cetuximab (CET) protein samples under non-reducing and reducing conditions. **(C)** Representative chromatograms of anti-EGFR/VEGF-A BsAb (15 µg) and faricimab (15 µg) protein samples were generated using SEC-HPLC method **(D)** Representative electropherograms of anti-EGFR/VEGF-A BsAb and faricimab protein samples were generated using non-reducing and reducing CE-SDS method. Data presented in this figure represented at least three independent experiments.

The strategy to generate an anti-EGFR/VEGF-A BsAb in a CrossMab/KIH format required the construction of four plasmids expressing the light chains and heavy chains of the BsAb for assembly (**Figure 1A**). The constructs were derived from cetuximab (CET) and faricimab (FAR) and were altered to produce anti-EGFR/VEGF-A BsAb. KIH technology ensured the heterodimerization of the heavy chains. Two heavy chains in the CH3 domain of the immunoglobulin Fc region contained “knob” and “hole” mutations. The “knob” mutation, T391W, replaced a smaller amino acid with the bulkier tryptophan on one heavy chain. On the second heavy chain, the corresponding “hole” mutations, T391S_L393A_Y432V, were generated to provide an opening with smaller amino acids that could sterically fit the “knob”. Thus, only heavy chains with “knob” mutations paired with heavy chains with “hole” mutations. CrossMab technology enabled the correct light chain association of the BsAb through domain crossovers. Our BsAb has a CrossMab^CH1-CL^ format that exchanged the CH1 and CL domain of the EGFR arm of cetuximab. The VEGF-A arm copied from faricimab was unaltered. The CH1-CL format was used to enforce the correct light chain association, provide structural stability, and theoretically yield no side product (16).

The structural integrity of the BsAb was evaluated using SDS-PAGE analysis. The anti-EGFR/VEGF-A BsAb, cetuximab, and faricimab displayed a ~150 kDa major band in non-reducing conditions (**Figure 1B**). There were faint bands present at lower molecular weight in all recombinant antibodies, indicating some degradation from boiling the protein samples at 95°C during sample preparation. In reducing conditions, two ~50 kDa heavy chain bands and two ~25 kDa light chain bands were observed for the anti-EGFR/VEGF-A BsAb. At the same molecular weight markers, three bands were observed for faricimab (two ~50kDa heavy chain bands and single ~25kDa light chain band), and two distinct bands were observed for cetuximab (**Figure 1B**). These data indicated that the anti-EGFR/VEGF-A BsAb contained heavy chains and light chains. Purity was evaluated with SEC-HPLC (**Figure 1C**) and CE-SDS analysis (**Figure 1D**). The recombinant antibodies had a single peak in the SEC-HPLC chromatograms without showing any signs of peaks at other migration times that would be associated with aggregation or fragmentation of the parent molecule. Faricimab and anti-EGFR/VEGF-A BsAb had different migrations times of 7.112 min and 6.832 min, respectively (**Figure 1C**). Further analysis of structural integrity and purity was performed with the CE-SDS measurements in non-reducing or reducing conditions. Non-reducing electrophoresis provided information on the structural integrity of the inter- and intrachain disulfides of the antibodies as a measure of purity (29–32). Purities in non-reduced conditions were determined as ~96.1% and ~73.8% for faricimab and anti-EGFR/VEGF-A BsAb, respectively. The second peak observed in the electropherogram of non-reduced anti-EGFR/VEGF-A BsAb at a lower molecular weight was consistent with either a native antibody subunit species or fragment that had separated from the intact BsAb or was caused by the denaturing conditions of non-reduced CE-SDS (30). Reducing conditions allowed the analysis of the relative chain distribution of the antibody into light chain (LC), heavy chain (HC), and non-glycosylated heavy chain components (NGHC) (29–32). Light chains in reduced conditions were observed as single peaks at ~1.2 RMT as 31.9% and 34.7% in faricimab and anti-EGFR/VEGF-A, respectively. The heavy chains had multiple peaks in reducing conditions. Faricimab contained two peaks at ~1.6 RMT and anti-EGFR/VEGF-A BsAb had three peaks at ~1.6-1.7 RMT (**Figure 1D**). The multiple peaks corresponded to the different molecular weights of the designed “knob” and “hole” heavy chains. The heavy chain distribution observed between faricimab and the anti-EGFR/VEGF-A BsAb varied. While faricimab had even distribution between its two heavy chain peaks at 32.5% and 35.6%, the anti-EGFR/VEGF-A BsAb had a more uneven distribution of 43.8% and 18.7% (**Figure 1D**). The third peak with a 2.8% area in the reduced CE-SDS chromatogram of the anti-EGFR/VEGF-A BsAb could arise from non-glycosylated heavy chain (**Figure 1D**). Both recombinant bispecific antibodies contained the same CrossMab/KIH format, however their CE-SDS electropherograms were different and unique to each protein sample. Overall, the anti-EGFR/VEGF-A BsAb maintained structural integrity and the purity was deemed to be adequate for further physiochemical characterization.

### 3.2 Binding characterization of anti-EGFR/VEGF-A BsAb

**Figure 2:**
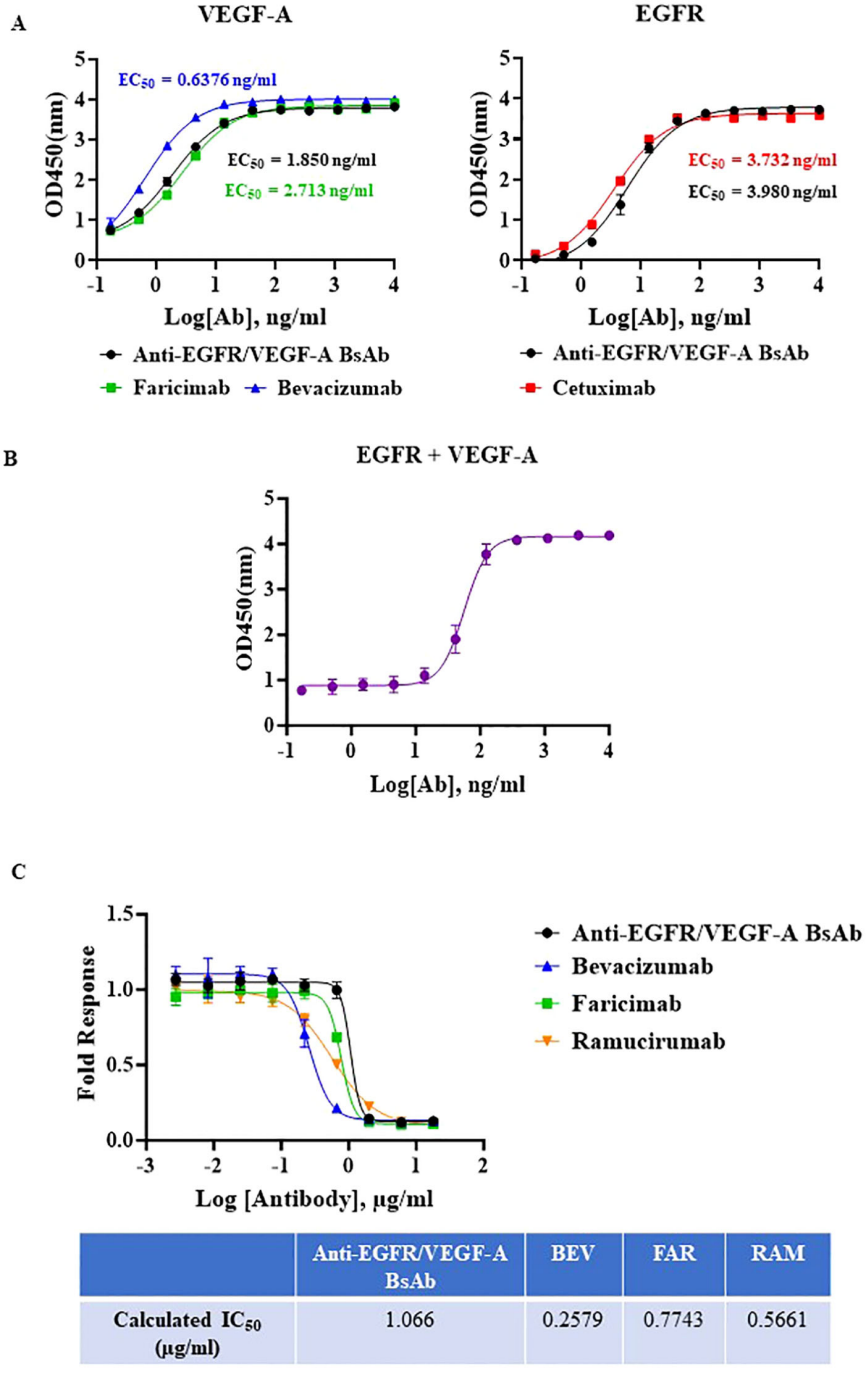
Potency characterization of anti-EGFR/VEGF-A BsAb. **(A)** Dose-dependent binding activity of anti-EGFR/VEGF-A BsAb, bevacizumab, faricimab, and cetuximab was evaluated using an ELISA binding assay. **(B)** Simultaneous binding activity of anti-EGFR/VEGF-A BsAb to its targets, biotinylated EGFR and VEGF-A was evaluated using an ELISA binding assay. **(C)** Dose-dependent inhibition of VEGF-A/VEGFR2 activation by anti-EGFR/VEGF-A BsAb, bevacizumab, faricimab, and ramucirumab was performed using a VEGF activity bioassay.

To evaluate binding ability of our anti-EGFR/VEGF-A BsAb, we performed enzyme-linked-immunosorbent-assay (ELISA) and surface plasmon resonance (SPR). A dose-dependent binding profile of anti-EGFR/VEGF-A BsAb toward recombinant VEGF-A and EGFR was evaluated by an ELISA method. The ELISA data confirmed that anti-EGFR/VEGF-A BsAb bound to VEGF-A and EGFR in a manner comparable to faricimab, bevacizumab, and cetuximab (**Figure 2A**). Anti-EGFR/VEGF-A BsAb had a calculated low EC_50_ value of 1.850 ng/mL for VEGF-A and 3.980 ng/mL for EGFR (**Figure 2A**). The data obtained from ELISA assay also showed that anti-EGFR/VEGF-A BsAb bound to EGFR and VEGF-A simultaneously with the EC_50_ value of 56.13 ng/mL (**Figure 2B**). Further confirmation of anti-EGFR/VEGF-A BsAb’s ability to bind to VEGF-A was shown using a VEGF-A bioassay. This functional assay contained a genetically engineered KDR/NFAT-RE HEK 293 cell line that luminesced upon VEGF-A binding to KDR (VEGFR2). Recombinant antibodies that target VEGF-A or VEGFR2 blocked luminescence. Anti-EGFR/VEGF-A BsAb bound to VEGF-A and blocked VEGFR2 activation in a dose-dependent manner, as did faricimab, bevacizumab, and ramucirumab (**Figure 2C**). To determine binding affinity, binding kinetics data were collected from SPR measurements using the Biacore T200 optical biosensor instrument (**Supplementary Figure 1**). Anti-EGFR/VEGF-A BsAb was immobilized on the Protein G Chip and serially diluted concentrations of solutions of recombinant VEGF-A and EGFR were flowed through the cells. Anti-EGFR/VEGF-A BsAb had strong binding affinities to both EGFR and VEGF-A that were similar to those of cetuximab and bevacizumab. The equilibrium dissociation constant (KD) value of anti-EGFR/VEGF-A BsAb to EGFR was 1.78x10–^9^ M (**Table 1**). We previously reported that the KD value of cetuximab to EGFR was 1.19x10^-9^ (27). The KD value of anti-EGFR/VEGF-A BsAb to VEGF-A was 1.72x10–^10^ M (**Table 1**). According to literature the K_D_ value of bevacizumab binding to VEGF-A was 5.8x10–^11^ M (33). Thus, SPR data showed that anti-EGFR/VEGF-A BsAb bound to VEGF-A and EGFR with comparable binding affinity to the parent mAbs.

**Table 1:** Binding kinetics parameters of anti-EGFR/VEGF-A BsAb to EGFR and VEGF-A were detected by a Biacore T200 optical biosensor instrument and analyzed using Biacore 8K Evaluation Software 3.0.

Ligand	ka (1/Ms)	kd (1/s)	KD (M)
EGFR	7.39x10^5^	1.31x10^-3^	1.78x10^-9^
VEGF-A	1.90x10^6^	3.25x10^-4^	1.72x10^-10^

### 3.3 Assessment of the thermal stability of anti-EGFR/VEGF-A

**Figure 3:**
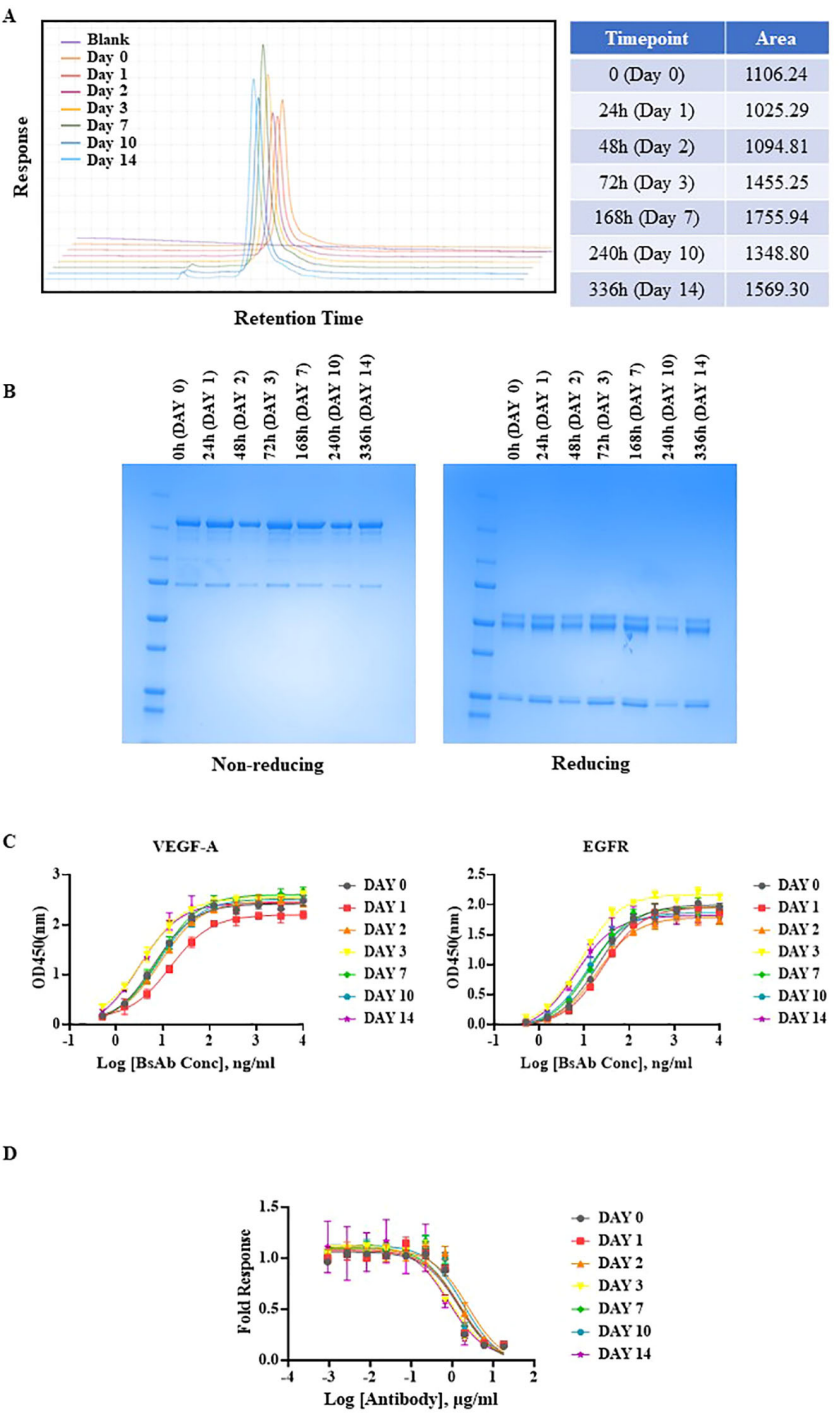
Thermal stability evaluation of anti-EGFR/VEGF-A BsAb. Anti-EGFR/VEGF-A BsAb (15 µg) was stressed at 42°C for 2 weeks. The protein samples were collected at seven different time points: 0 h (Day 0), 24 h (Day 1), 48 h (Day 2), 72 h (Day 3), 168 h (Day 7), 240 h (Day 10), and 336 h (Day 14). **(A)** Overlaid chromatograms of thermal stressed and unstressed anti-EGFR/VEGF-A BsAb protein samples was generated using a SEC-HPLC method. **(B)** SDS-PAGE analysis showed the structural integrity of thermal stressed and unstressed anti-EGFR/VEGF-A BsAb protein samples under non-reducing and reducing conditions. **(C)** Dose-dependent binding activity of anti-EGFR/VEGF-A BsAb protein samples to EGFR and VEGF-A was evaluated using the ELISA binding assay. **(D)** Dose-dependent inhibition of VEGF-A/VEGFR2 activation by thermal stressed and unstressed anti-EGFR/VEGF-A BsAb protein samples was performed using a VEGF activity bioassay.

Next, the anti-EGFR/VEGF-A BsAb was subjected to both a short-term and prolonged thermal stress testing. The initial short-term thermal stress test consisted of 30 min of thermal stress at 25°C, 37°C, 42°C, 56°C, and 95°C in storage buffer PBS at pH 7.2 (**Supplementary Figure 2**). SEC-HPLC indicated degradation of our BsAb at 56°C and 95°C (**Supplementary Figure 2**). For a long-term thermal stress test, 42°C was chosen to evaluate the stability of our BsAb. The anti-EGFR/VEGF-A BsAb was subjected to thermal stress at 42°C for 0 h, 24 h, 48 h, 72 h, 168 h, 240 h, and 336 h in storage buffer at PBS at pH 7.2. The thermal stability of the protein samples was analyzed using SEC-HPLC and SDS-PAGE methods. A single peak was observed in all samples in their chromatograms (**Figure 3A_graph**). However, the peak areas from samples collected at the different time points varied (**Figure 3A_table**). The variability in peak area was attributed to samples being collected on the different days. Nevertheless, the 168 h, 240 h and 336 h sample chromatograms showed less than 2% peak areas from new peaks relative to the main peak (attributed to protein aggregates). Non-reducing and reducing SDS-PAGE conditions supported the conclusion that the BsAb samples remained stable at all timepoints (**Figure 3B**). The stability of the higher order structure of anti-EGFR/VEGF-A BsAb was similar to stability characteristics observed for the anti-EGFR/CD3 BsAb in DVD-IgG format. This similarity was attributed to both molecular formats containing an Fc domain, unlike a BsAb that does not have the Fc portion, such as BiTE (34). While structurally the anti-EGFR/VEGF-A BsAb remained intact following the thermal stresses, it was also important to confirm whether or not the thermal stress affected its function. To elucidate the potential for the thermal stress to impact binding, an ELISA assay was performed. Compared to the unstressed sample at 0 h, the stressed samples had comparable binding activity (**Figure 3C**). Calculated EC_50_ values remained constant at all timepoints (**Supplementary Table 1**), confirming that thermal stress did not impede the BsAb’s ability to bind to VEGF-A and EGFR. With binding activity still intact, the stressed protein samples retained the ability to block VEGFR2 activation through VEGF-A ligand binding. Inhibition of VEGF-A binding to KDR was exhibited by all stressed protein samples in a dose dependent manner, similar to the unstressed sample (**Figure 3D**). Therefore, thermal stress did not observably degrade the structural integrity and the desired pharmacological functions of anti-EGFR/VEGF-A BsAb.

### 3.4 Mechanistic inhibition of EGFR and VEGFR2 activation in an ovarian cancer cell model

**Figure 4:**
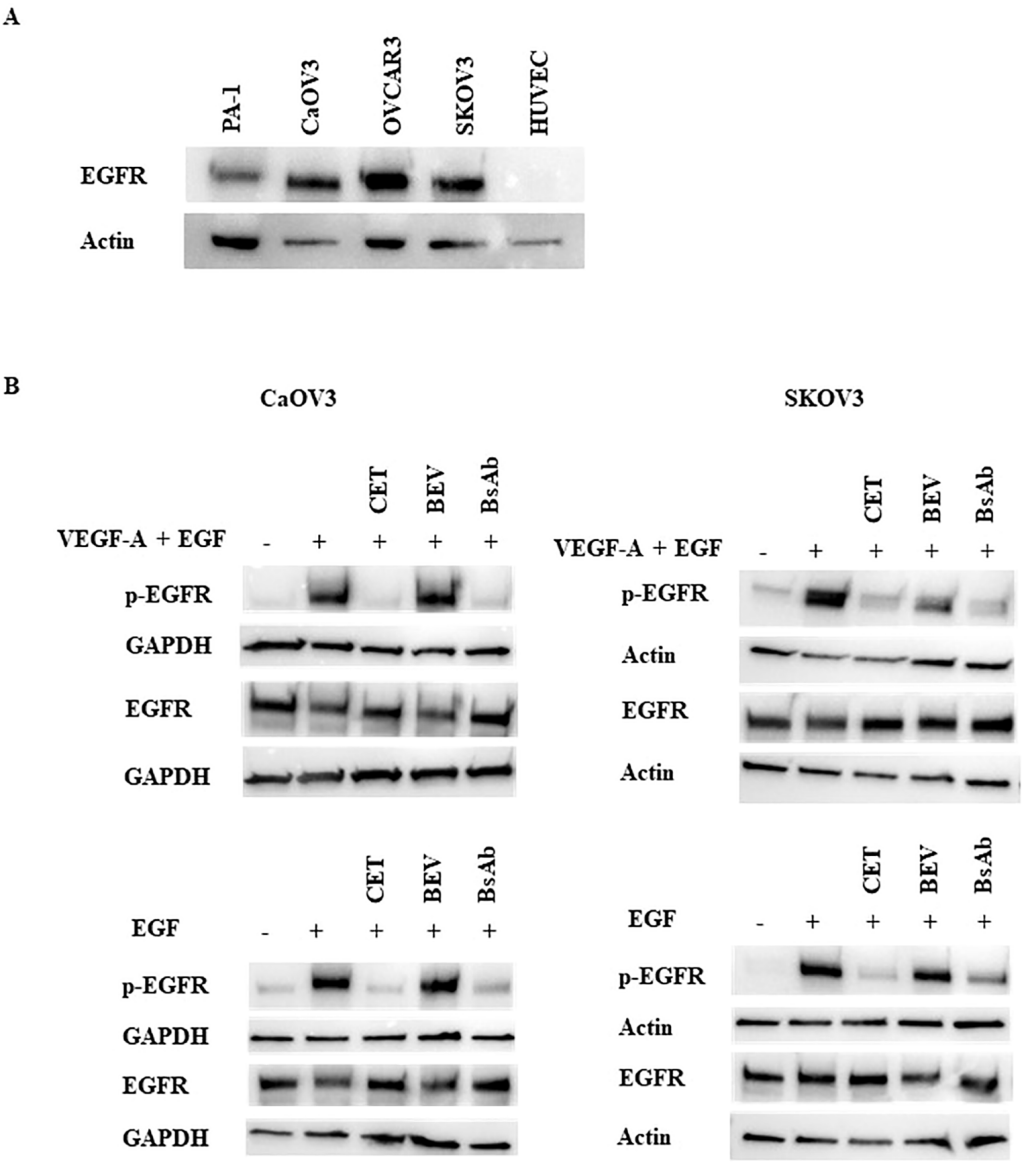
Inhibition of ligand-induced activation of EGFR by anti-EGFR/VEGF-A BsAb. **(A)** Western blot analysis was performed to measure EGFR expression levels in HUVEC and OC cell lines: PA-1, CaOV3, OVCAR3, and SKOV3 cells. Whole cell lysates were prepared from each cell line, and Western blotting was performed to measure relative EGFR protein levels in these cell lines. **(B)** Phospho-EGFR and EGFR levels were measured using Western blot analysis of serum-starved WCL collected from CaOV3 and SKOV3 cells. Cells were pre-treated with 10 µg indicated monoclonal antibodies and BsAb for 1 hour followed by 100 ng/mL ligand stimulation for 15 minutes.

**Figure 5:**
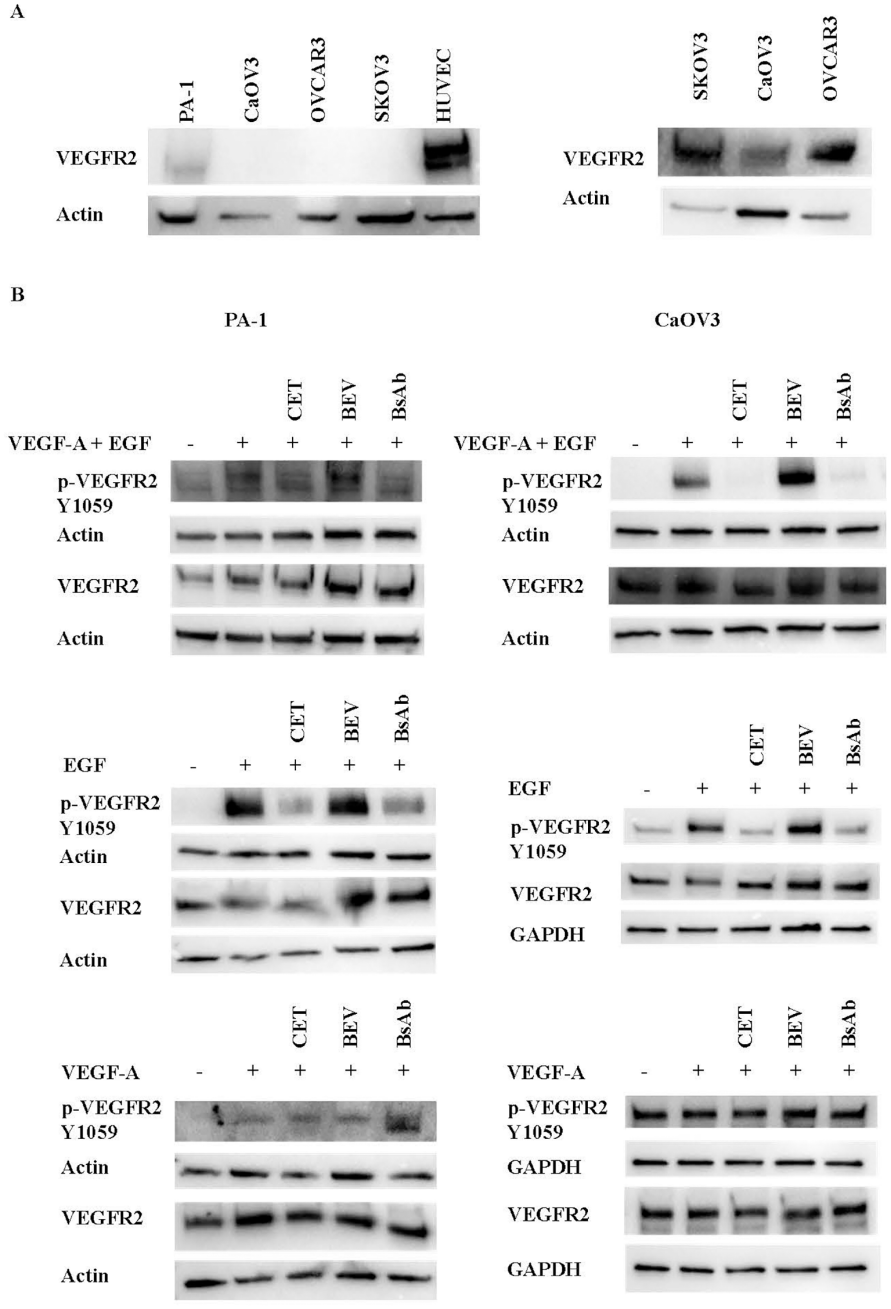
Inhibition of ligand-induced activation of VEGFR2 by anti-EGFR/VEGF-A BsAb. **(A)** Western blot analysis was performed to measure VEGFR2 expression levels in HUVEC and OC cell lines: PA-1, CaOV3, OVCAR3, and SKOV3 cells. WCL were prepared from each cell line, and Western blot was performed to measure relative VEGFR2 protein levels in these cell lines. **(B)** Phospho-VEGFR2 and VEGFR2 levels were measured using western blot analysis of serum-starved WCL collected from CaOV3 and PA-1 cells. Cells were pre-treated with 10 µg indicated monoclonal antibodies and BsAb for 1 hour followed by 100 ng/mL ligand stimulation for 15 minutes.

To examine the mechanism of action of the anti-EGFR/VEGF-A BsAb in an ovarian cancer (OC) cell model, the EGFR and VEGFR2 signaling pathways were studied. Expression analysis of EGFR levels in a panel of OC and HUVEC cells showed that SKOV3, OVCAR3, CaOV3, and PA-1 cells expressed EGFR at varying expression levels (**Figure 4A**). OVCAR3, SKOV3, and CaOV3 had higher EGFR expression compared to PA-1 cells, while HUVEC cells did not express measurable EGFR (**Figure 4A**). Anti-EGFR/VEGF-A BsAb inhibited ligand-induced EGFR activation in SKOV3 and CaOV3 cells (**Figure 4B**). EGF binds to the extracellular domain of EGFR, inducing dimerization that draws the intracellular kinase domains close enough so that trans-autophosphorylation can occur (35). Treatments of combined ligands (EGF + VEGF-A) and EGF alone increased the phosphorylation of EGFR at the Y1086 phosphorylation site in CaOV3 and SKOV3 cells (**Figure 4B**). Anti-EGFR/VEGF-A BsAb and cetuximab (CET) blocked EGFR phosphorylation through competitively binding to EGFR and inhibited EGF-induced EGFR phosphorylation (**Figure 4B**). Bevacizumab (BEV) did not block EGF-induced EGFR phosphorylation (**Figure 4B**). PA-1, SKOV3, CaOV3, OVCAR3, and HUVEC cells expressed VEGFR2 at varying amounts (**Figure 5A**, **Supplementary Figure 3**). HUVEC cells had the highest expression of VEGFR2 (**Figure 5A**, **Supplementary Figure 3**). Due to amounts of VEGFR2 expression in HUVEC cells, we separately examined the expression of VEGFR2 in SKOV3, CaOV3, OVCAR3 cells using SDS-PAGE (**Figure 5A** left panel). In a similar manner as EGF-EGFR, VEGF-A is known to activate the VEGFR2 pathways in OC cells (24, 36, 37). Binding of VEGFs to VEGFRs induces receptor homo- or hetero-dimerization, leading to autophosphorylation of the tyrosine residues (24, 36, 38). To investigate these signal transduction pathways, phosphorylation of VEGFR2 at the Y1059 site in PA-1 and CaOV3 cells was measured. Similar to EGFR activation, combined ligands (EGF + VEGF-A) or EGF alone activated VEGFR2 phosphorylation at the Y1059 site in CaOV3 and PA-1 cells. Interestingly, EGF appeared to activate the phosphorylation of VEGFR2 in CaOV3 and PA-1 cells, potentially indicating cross-talk between EGFR and VEGFR2 signal transduction pathways in these OC cells (**Figure 5B**). The anti- EGFR mAb, cetuximab (CET), inhibited the ligand-induced phosphorylation of VEGFR2 at the Y1059 site, as did the anti-EGFR/VEGF-A BsAb. Bevacizumab (BEV) did not block the Y1059 phosphorylation of VEGFR2 induced by VEGF-A + EGF and EGF. Conversely, it was reported that that EGFR and VEGFR2 did interact in the absence of ligand or in the presence of both ligands using a qualitative FRET study that monitored EGFR and VEGFR2 in the plasma membrane of live HEK293 cells (36). However, in the case of CaOV3 cells, a co-immunoprecipitation study showed that VEGFR2, and EGFR did not interact in the presence of both ligands (**Supplementary Figure 4**), indicating that EGF-induced activation of VEGFR2 was mediated by the downstream of receptors through cross-talking pathways. Additionally, VEGF-A did not sufficiently activate VEGFR2 in CaOV3 or PA-1 cells (**Figure 5B**). The ADCC activity was not detected for anti-EGFR/VEGF-A BsAb (**Supplementary Figure 5**). Taken together, these results suggest that there are complex signaling pathways mediated by EGFR and VEGFR2 in OC cells, and the activation of VEGFR2 may occur through EGFR in OC cells.

### 3.5 Disruption of paracrine VEGFR2 activation in HUVEC cells

**Figure 6:**
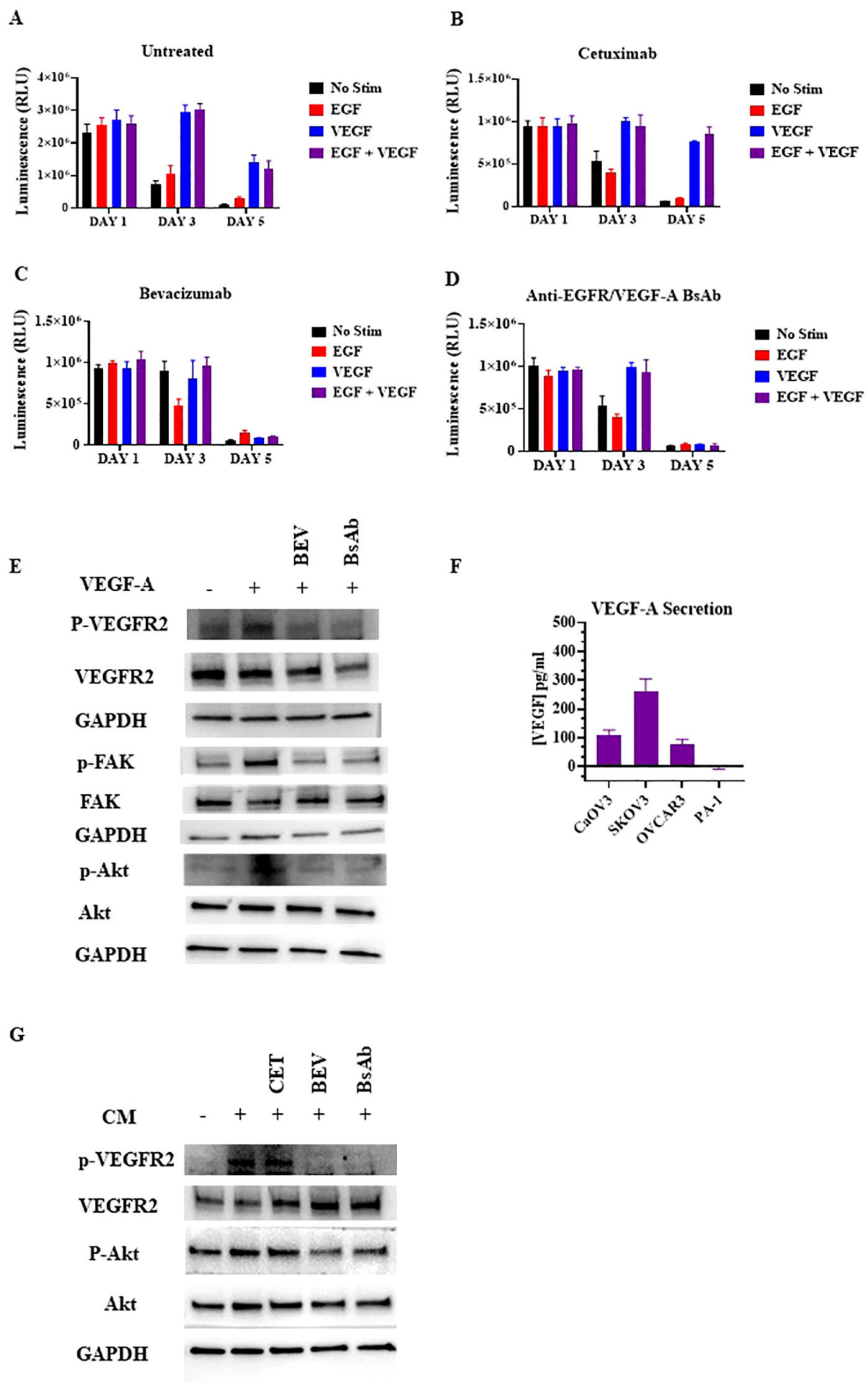
Inhibition of paracrine VEGFR2 activation in HUVECs by anti-EGFR/VEGF-A BsAb. **(A)** The CellTiter-Glo luminescent cell viability assays were performed in HUVEC cells. 10,000 cells were seeded in white bottom of 96-well plates and allowed to adhere overnight in media with 1% FBS. After treatments with 10 μg/mL cetuximab **(B)**, bevacizumab **(C)**, or anti-EGFR/VEGF-A BsAb **(D)**, CellTiter-Glo reagent was added into the plates and luminescence (i.e., viability) was measured using a Promega GloMax Discover plate reader. **(E)** Western blot analysis was performed to measure the inhibition of ligand-induced activation of VEGFR2 and its downstream pathways (Akt, and FAK) by bevacizumab (BEV) and anti-EGFR/VEGF-A BsAb. **(F)** The levels of VEGF-A were determined by ELISA assay in supernatants of CaOV3, SKOV3, OVCAR3, and PA-1 cells after serum-starving the cells in a 6-well-plate for 48 h **(G)** Inhibition of the conditional media (CM)-mediated VEGFR2 activity in HUVEC cells by cetuximab (CET), bevacizumab (BEV), and anti-EGFR/VEGF-A BsAb. The conditional media (CM) samples were collected from SKOV3 cell culture after 48 h serum-starvation. HUVEC cells were serum-starved for 24 h CM samples were collected from the SKOV3 cells and pre-incubated with 10 μg/mL cetuximab (CET), bevacizumab (BEV), or anti-EGFR/VEGF-A BsAb. HUVEC media was removed from the cells and replaced with the CM for 2 h before WCL was harvested. Whole cell lysates were then subjected to western blot analysis.

VEGFR2 can be activated through paracrine activation (24, 37, 38). The VEGFR2 expression amounts observed in HUVEC cells make these cells an ideal model cell line to explore the paracrine VEGFR2 signaling pathway between OC and endothelial cells. To evaluate the effect of VEGF-A stimulation on HUVEC cells, cell viability assays were performed (**Figure 6A**). HUVEC cells were incubated with 100 ng/mL VEGF-A and/or 100 ng/mL EGF. Cell viability was assessed at different incubation timepoints: day 1, day 3, and day 5. VEGF-A alone or combined with EGF promoted cell survival in HUVEC cells cultured in low serum at the day 5 timepoint (**Figure 6A**). The addition of bevacizumab and anti-EGFR/VEGF-A BsAb, but not addition of anti-EGFR antibody (cetuximab), reversed the protective effect of VEGF-A with or without EGF on the HUVEC cells (**Figures 6B–D**). Western blot analysis of the VEGFR2 signaling pathway confirmed that VEGF-A activated VEGFR2 and its downstream signaling pathway, including focal adhesion kinase (FAK) and Akt in HUVEC cells (**Figure 6E**). Bevacizumab (BEV) and anti-EGFR/VEGF-A BsAb blocked phosphorylation of the Y1054 site of VEGFR2 and subsequently blocked downstream signaling pathways (**Figure 6E**).

In the paracrine model, OC cells secrete VEGF-A to activate VEGFR2 signaling in endothelial cells promoting tumor growth and metastasis (24, 37–39). A VEGF ELISA method was performed to identify and quantify which OC cell lines secretes VEGF-A. OC cell lines were serum-starved for 48 h and then, the conditional cell media was collected and tested. SKOV3, OVCAR3, and CaOV3 cells secreted varying amounts of VEGF-A, while no detectable VEGF-A was observed in PA-1 cells(**Figure 6F**). CaOV3 and OVCAR3 secreted ~100 pg/mL VEGF-A, while SKOV3 secreted ~300 pg/mL in 200 uL of conditional cell media (**Figure 6F**). To determine whether conditional media containing secreted VEGF-A from OC cells could activate the VEGFR2 in HUVEC cells, potentially via a paracrine mechanism, Western blot analysis was used. SKOV3 cells were serum starved and pre-treated with indicated antibodies for 48 h. HUVEC cells were treated with the conditional media from the SKOV3 cells for 15 min and lysed for Western blot analysis. The secreted VEGF-A from SKOV3 induced VEGFR2 phosphorylation at the Y1059 site in HUVEC cells, and bevacizumab and the anti-EGFR/VEGF-A BsAb blocked phosphorylation at the Y1059 site (**Figure 6G**). These data support the conclusion that anti-EGFR/VEGF-A BsAb inhibits the paracrine activation of VEGFR2 in OC.

## 4 Discussion

The production of a single BsAb, as opposed to two or more mAbs, allows for simpler, streamlined manufacturing with reduced cost (15). BsAbs can be manufactured in a steady, reproducible fashion at a large-scale, while meeting quality aspects, including during upstream and downstream processing (16). Using CrossMab technology together with KIH technology, we designed and generated an anti-EGFR/VEGF-A BsAb that had the desired stability. Our BsAb shared the same molecular format as faricimab, with some differences in design. A side-by-side comparison of our BsAbs with faricimab using methods commonly used for the characterization of therapeutic monoclonal antibodies and BsAbs showed similar quality attribute values.

In this study the heavy and light chains of the BsAb were successfully expressed in HEK 293 cells using the transient expression approach to produce an anti-EGFR/VEGF-A BsAb for laboratory research purposes, and subsequent physicochemical characterization supported accurate BsAb assembly. Compared to the mAbs bevacizumab and cetuximab, anti-EGFR/VEGF-A BsAb exhibited a comparable, but observably lower binding affinity to target antigens. A decrease in BsAb binding avidity was also reflected in the VEGF secretion bioassay, as the calculated IC_50_ values for the mAbs were lower than the BsAb. However, a noticeable difference in the profiles of non-reduced CE-SDS between our BsAb and faricimab was observed. This could reflect the impact of product-related impurities that were not detected by SEC-HPLC. Another potential cause of this difference could be disulfide scrambling occurring under the denaturing conditions of non-reduced CE-SDS, leading to artificial fragmentation (30, 40). Nevertheless, orthogonal methods used to detect potential product-related impurities are essential. Another advantage seen in the molecular format of our BsAb is stability under stressed conditions. Thermal stress experiments showed the structural integrity of our anti-EGFR/VEGF-A BsAb was retained a with no observed impact on binding function.

BsAbs, based on the mechanisms of action, may be broadly categorized as combinatorial mode or obligate mode. Combinatorial mode refers to combination of the activity of two antibodies into one molecule, whereas obligate mode combines two antigen binding sites to creates a temporal or spatial activity (41). Similar to the mode of action of faricimab (41), our BsAb showed a combinatorial mode of action demonstrating dual potency that inhibited VEGF-A-mediated activity and also blocked EGF/EGFR signaling pathways. We established an ELISA to show simultaneously binding of our BsAb to EGFR and VEGF-A. However, it has been difficult to develop one bioassay capable of assessing the potency of both arms of our BsAb. Thus, two separate bioassays reflecting the MOAs of BsAb with combinatorial mode may be necessary for product characterization and potentially for product lot release and stability testing.

Our BsAb was created under the premise that EGFR and VEGF-A would be effective therapeutic targets, as they modulate pathways that promote tumor growth, angiogenesis, and metastasis in OC. Studies have shown that EGFR expression is connected to poorer prognosis and decreased therapeutic response (21). Likewise, overexpressed VEGF-A/VEGFR2 has been observed in OC (24). Studies indicate the EGFR and VEGFR2 pathways have crosstalk that promote the resistance of single agent therapies (39). Our BsAb inhibited EGF-induced activation of EGFR in CaOV3 and SKOV3, as well as blocked EGF-induced activation of VEGFR2 in CaOV3 and PA-1 OC cancer cell lines. We hypothesize that EGF has a role in the VEGFR2 pathway in these specific OC cell lines. While our findings highlight the need to understand the role EGFR and VEGFR2 crosstalk plays in OC, this study also revealed a paracrine mechanism by which OC cells could activate angiogenic pathways in endothelial cells, which further support OC progression. Targeting both EGFR and VEGF-A via a bispecific antibody binding potentially enhances anti-tumor activity not only by inhibiting EGFR signaling in OC, but also by blocking angiogenic activity in endothelial cells that support tumor growth and progression.

## Data Availability

The original contributions presented in the study are included in the article/**Supplementary Material**. Further inquiries can be directed to the corresponding author.

## Supplementary material

Supplementary Figure 1 Biacore SPR electrogram binding activities of BsAb. Surface plasmon resonance (SPR) showed the binding kinetics of anti-EGFR/VEGF-A BsAb to EGFR and VEGF-A as detected by a Biacore T200 optical biosensor.

Supplementary Figure 2 Short term-thermal stress evaluation of Anti-EGFR/VEGF-A BsAb. 15 µg Anti-EGFR/VEGF-A BsAb was thermal stressed at 25°C, 37°C, 42°C, 56°C, 95°C for 30 min. Overlaid chromatogram of thermal stressed anti-EGFR/VEGF-A BsAb protein samples was generated using SEC-HPLC method.

Supplementary Figure 3 VEGFR2 expression in OC cell lines. Western blot analysis was performed to measure VEGFR2 expression levels in OC cell lines: PA-1, CaOV3, OVCAR3, and SKOV3. WB analysis of WCL was prepared from each cell line.

Supplementary Figure 4 Co-immunoprecipitation in CaOV3 cells. Whole cell lysates from CaOV3 cells were subjected to co-immunoprecipitation assay to determine the association of EGFR with VEGFR2. The indicated anti-EGFR monoclonal antibodies and BsAb were immunoprecipitated from WCL using Protein A and G beads, and WB analysis were performed to detect the immunoprecipitated EGFR and co-immunoprecipitated VEGFR2.

Supplementary Figure 5 Evaluation of anti-EGFR/VEGF-A-mediated antibody dependent cell-mediated cytotoxicity (ADCC) activity. CaOV3, SKOV3, and PA-1 cells were used for the assay. ADCC activity was determined using ADCC Report Bioassay.

Supplementary Table 1 EC50 values of ELISA binding activity. Calculated EC_50_ values of ELISA binding activity to VEGF-A and EGFR from thermal stressed and unstressed anti-EGFR/VEGF-A BsAb protein samples were generated from GraphPad Prism analysis.
