# Supplementary figures and images for: Physicochemical and biological characterization of a bispecific antibody in a CrossMab/KIH format that targets EGFR and VEGF-A
Source: Front Immunol. 2025 Sep 3;16:1659966. doi: 10.3389/fimmu.2025.1659966 (PMC12441059; doi:10.3389/fimmu.2025.1659966)

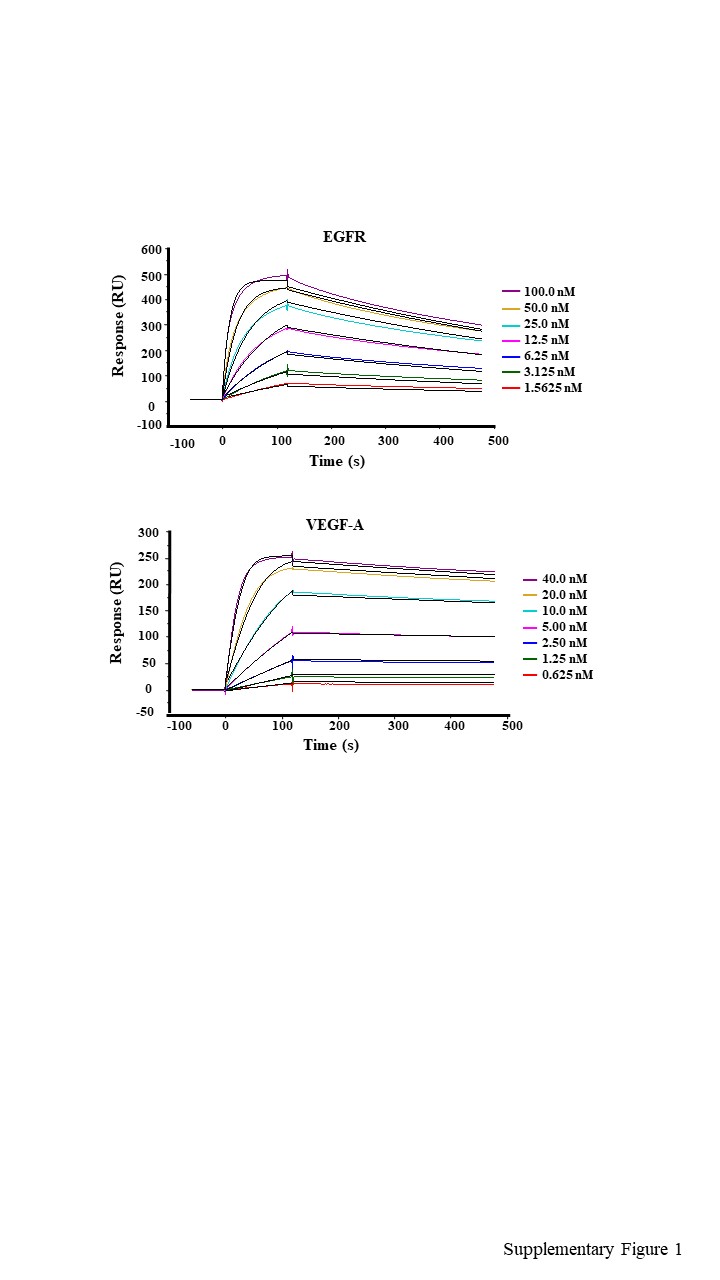

Supplement: Supplementary Figure 1 — Biacore SPR electrogram binding activities of BsAb. Surface plasmon resonance (SPR) showed the binding kinetics of anti-EGFR/VEGF-A BsAb to EGFR and VEGF-A as detected by a Biacore T200 optical biosensor. [file Image1.jpeg]

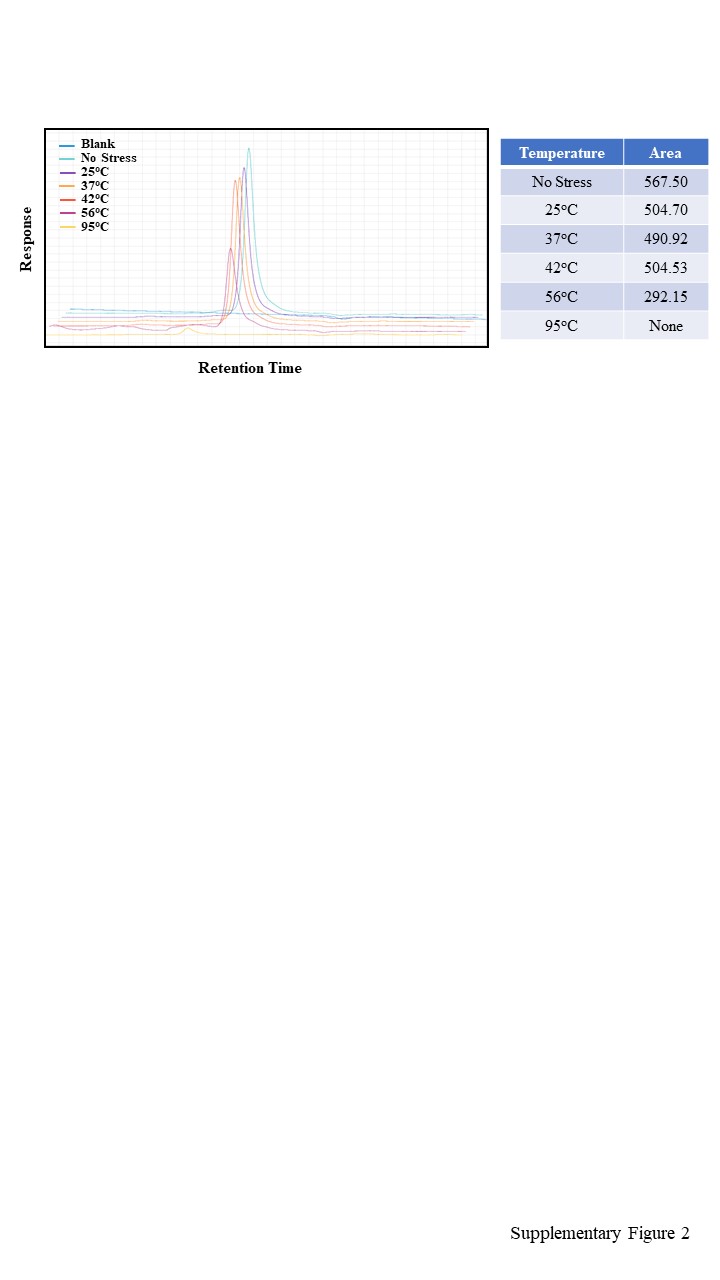

Supplement: Supplementary Figure 2 — Short term-thermal stress evaluation of Anti-EGFR/VEGF-A BsAb. 15 µg Anti-EGFR/VEGF-A BsAb was thermal stressed at 25°C, 37°C, 42°C, 56°C, 95°C for 30 min. Overlaid chromatogram of thermal stressed anti-EGFR/VEGF-A BsAb protein samples was generated using SEC-HPLC method. [file Image2.jpeg]

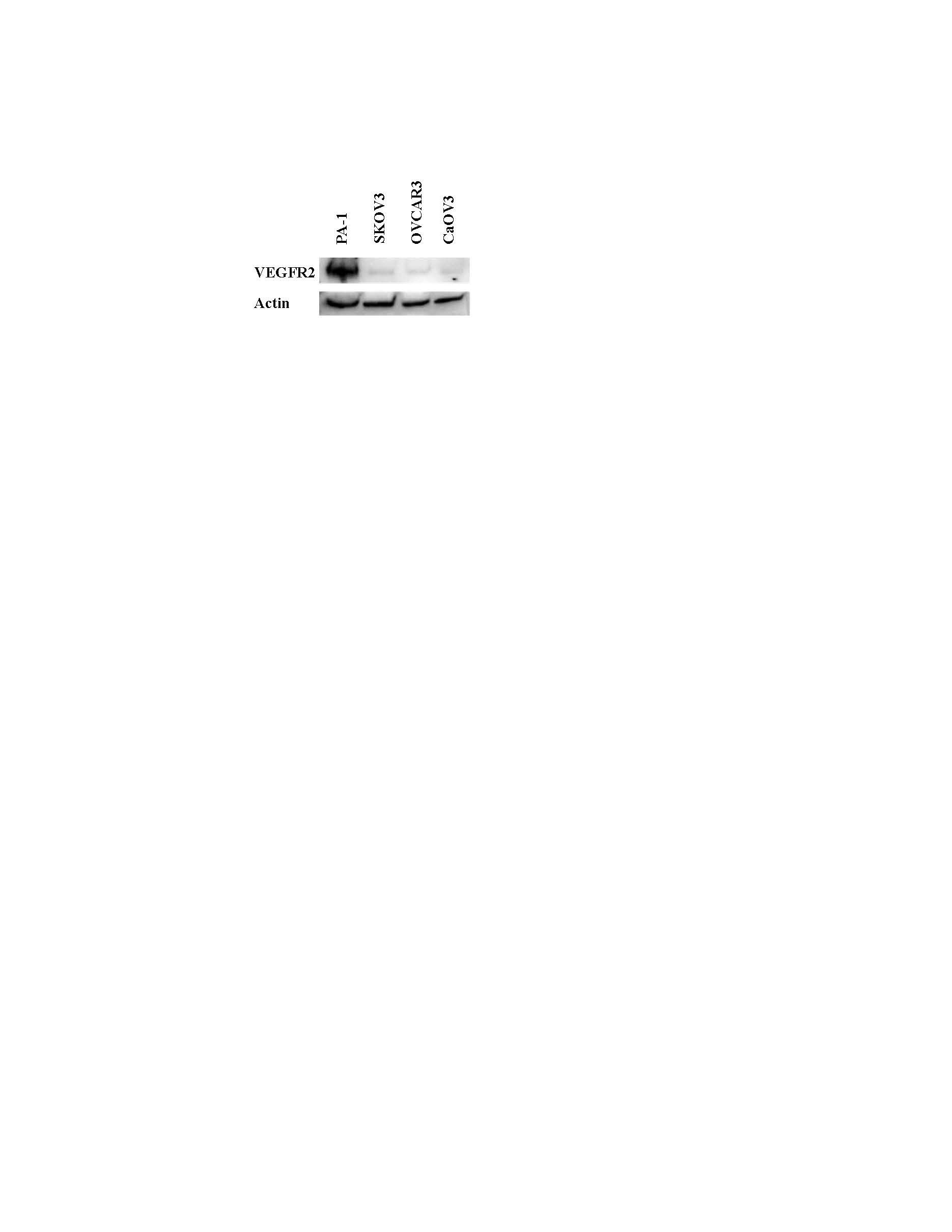

Supplement: Supplementary Figure 3 — VEGFR2 expression in OC cell lines. Western blot analysis was performed to measure VEGFR2 expression levels in OC cell lines: PA-1, CaOV3, OVCAR3, and SKOV3. WB analysis of WCL was prepared from each cell line. [file Image3.jpg]

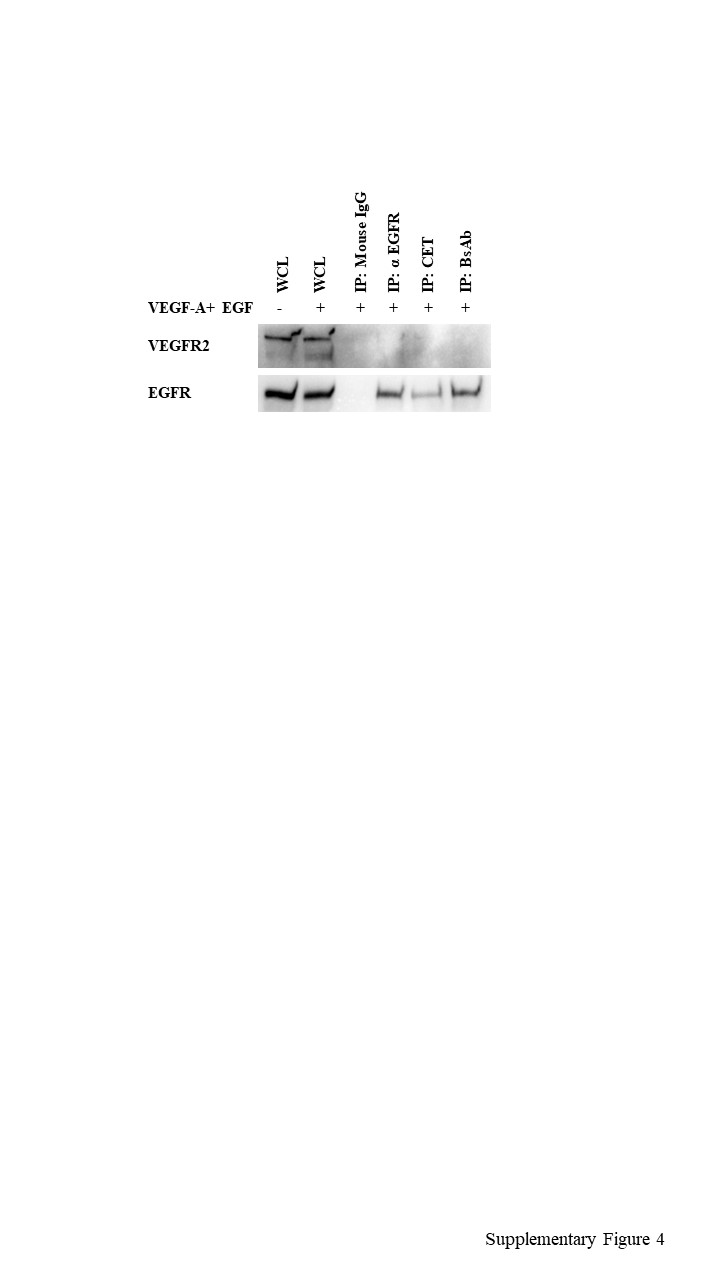

Supplement: Supplementary Figure 4 — Co-immunoprecipitation in CaOV3 cells. Whole cell lysates from CaOV3 cells were subjected to co-immunoprecipitation assay to determine the association of EGFR with VEGFR2. The indicated anti-EGFR monoclonal antibodies and BsAb were immunoprecipitated from WCL using Protein A and G beads, and WB analysis were performed to detect the immunoprecipitated EGFR and co-immunoprecipitated VEGFR2. [file Image4.jpeg]

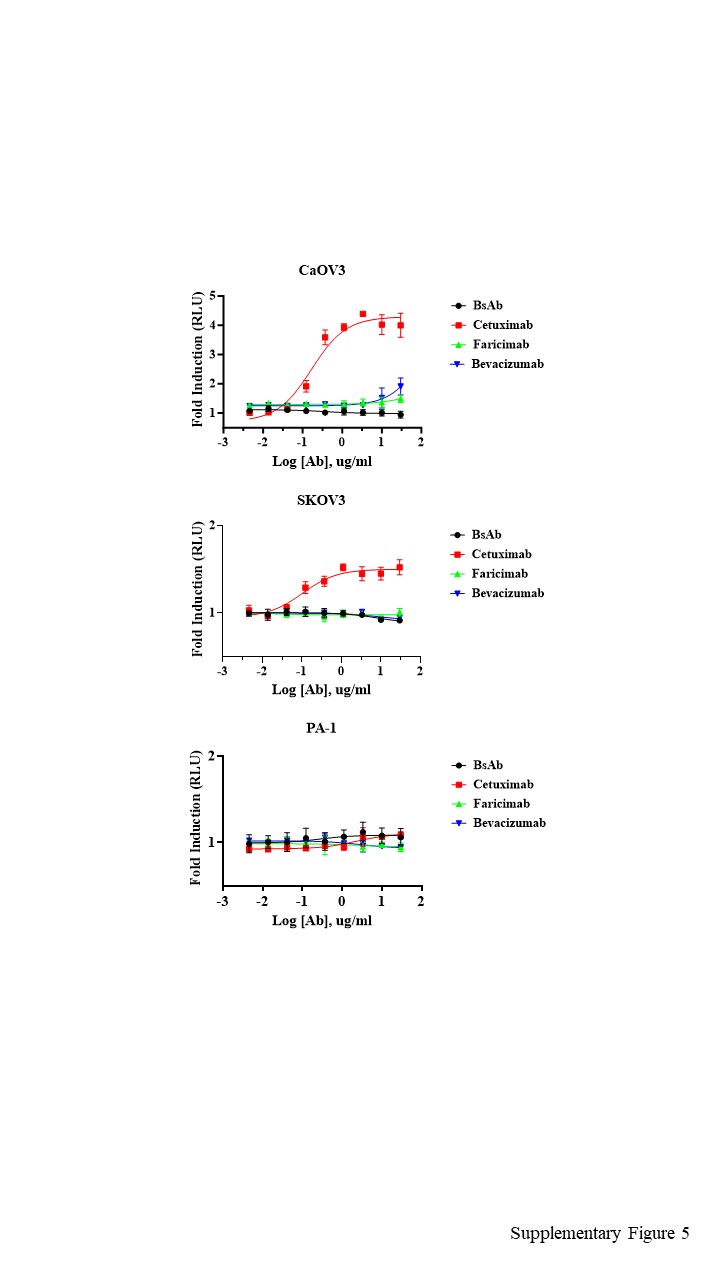

Supplement: Supplementary Figure 5 — Evaluation of anti-EGFR/VEGF-A-mediated antibody dependent cell-mediated cytotoxicity (ADCC) activity. CaOV3, SKOV3, and PA-1 cells were used for the assay. ADCC activity was determined using ADCC Report Bioassay. [file Image5.jpeg]

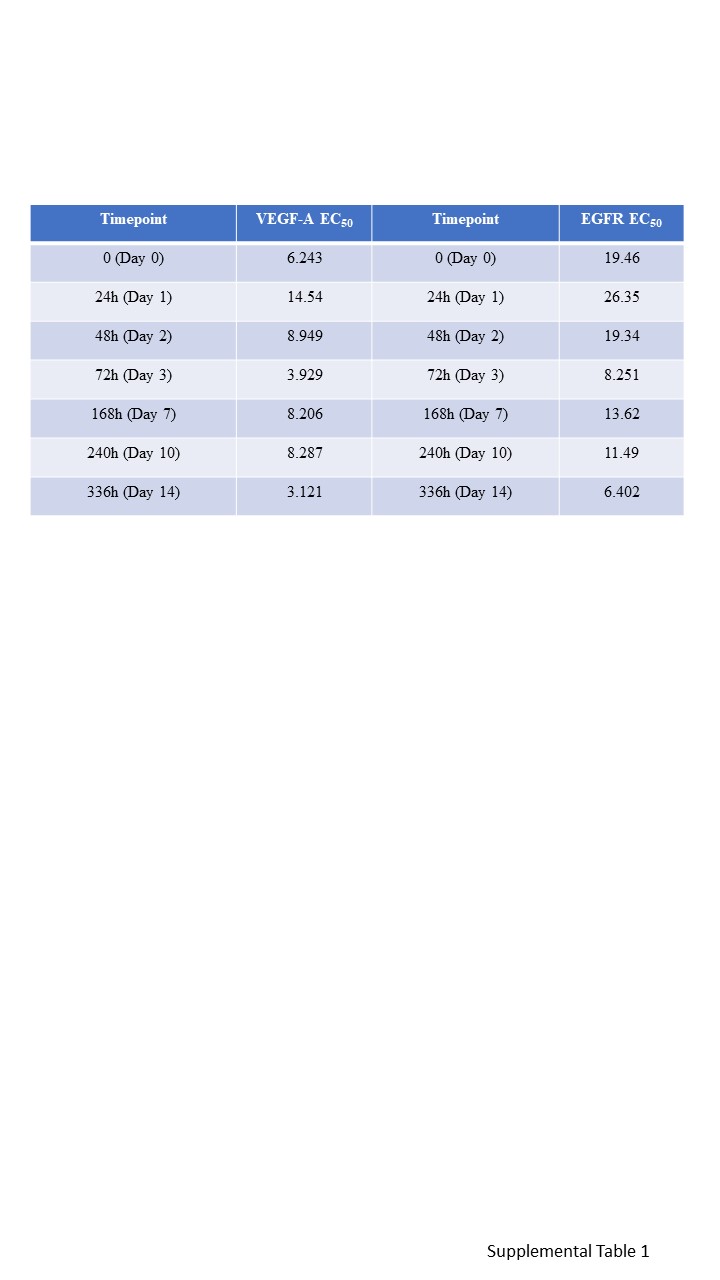

Supplement: Supplementary Table 1 — EC50 values of ELISA binding activity. Calculated EC50 values of ELISA binding activity to VEGF-A and EGFR from thermal stressed and unstressed anti-EGFR/VEGF-A BsAb protein samples were generated from GraphPad Prism analysis. [file Image6.jpeg]
